# Supplementary figures and images for: Crystal structure of Mokola virus glycoprotein in its post-fusion conformation
Source: PLoS Pathog. 2020 Mar 9;16(3):e1008383. doi: 10.1371/journal.ppat.1008383 (PMC7082061; doi:10.1371/journal.ppat.1008383)

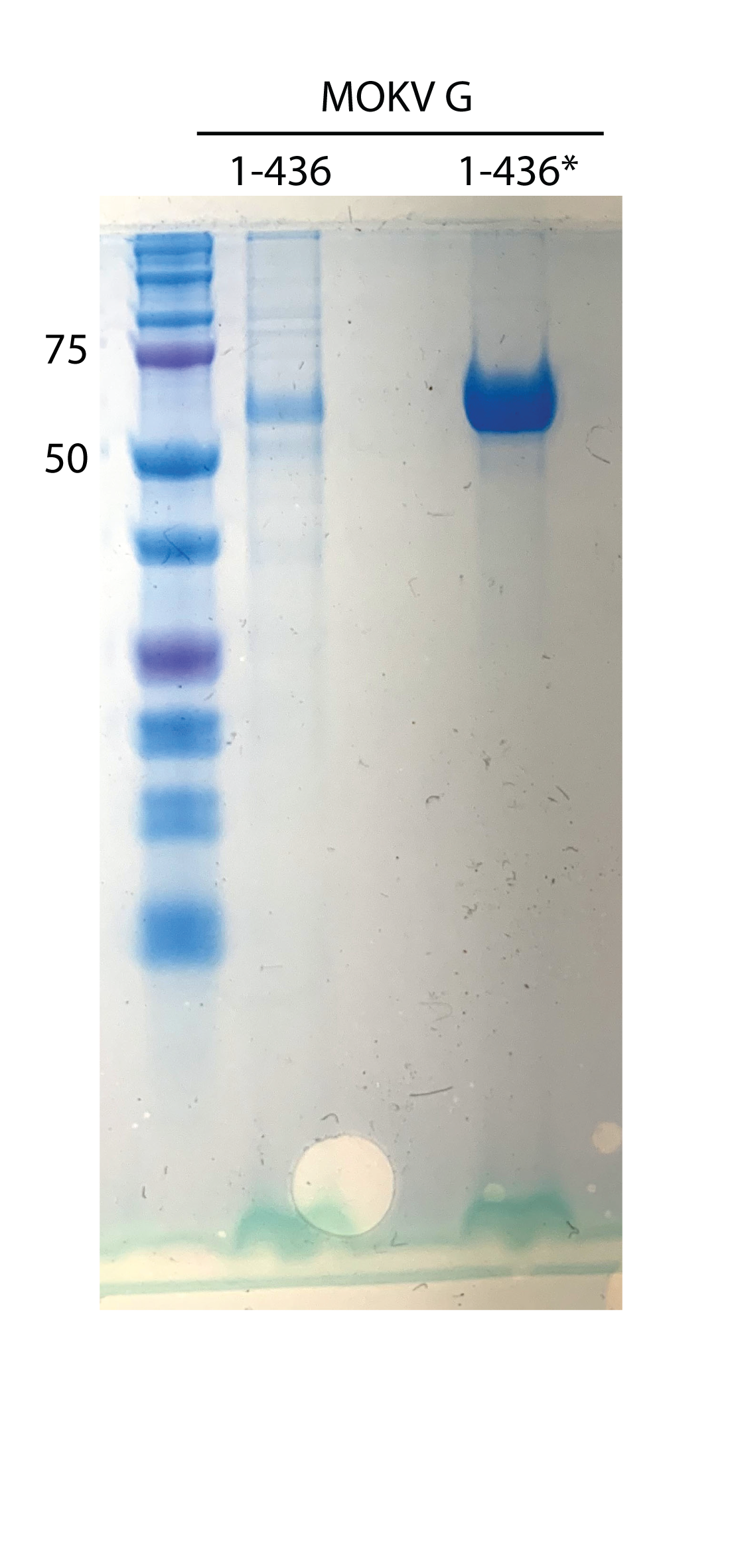

Supplement: S1 Fig — (TIF) [file ppat.1008383.s001.tif]

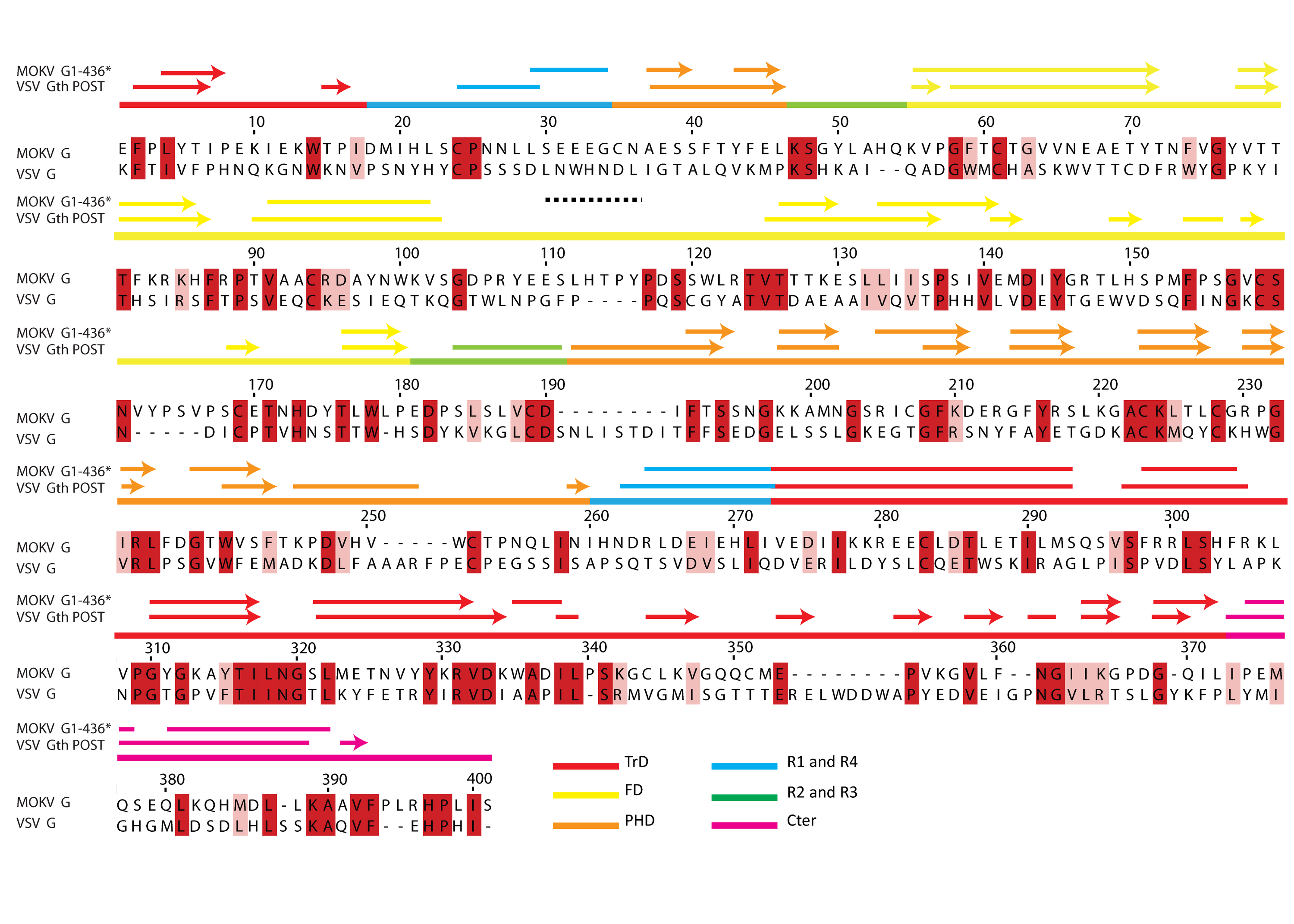

Supplement: S2 Fig — Conserved residues are in red boxes, whereas similar ones are in pink. Domains are identified by colors following Table 2 color code. Secondary structures of MOKV G and VSV G post-fusion state are indicated by arrows for β-sheets and straight lines for helices. (TIF) [file ppat.1008383.s002.tif]

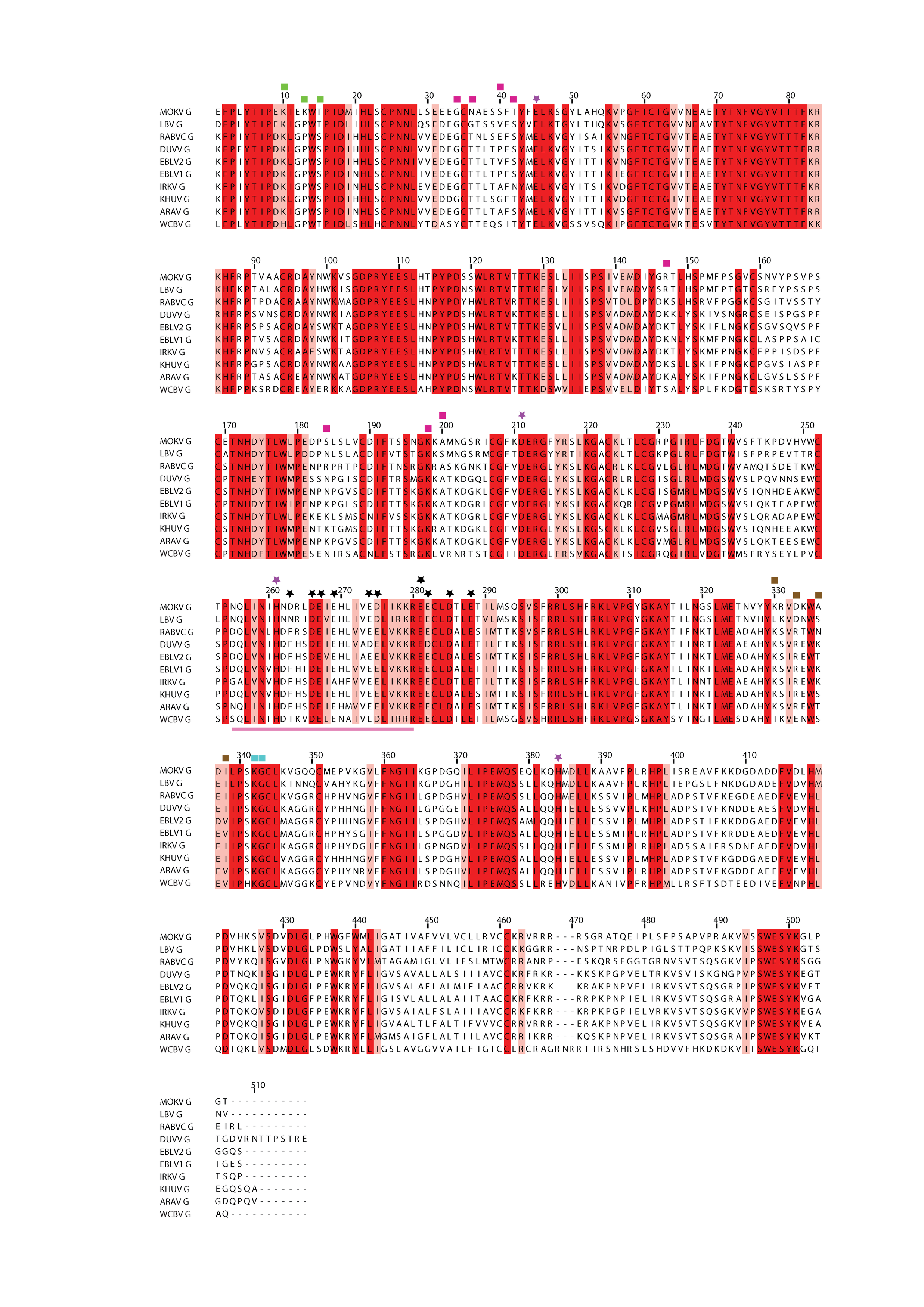

Supplement: S3 Fig — Conserved residues are in red boxes, whereas similar ones are in pink. A square indicates residues that are found substituted in mutants escaping neutralization by MAbs (in magenta for antigenic site II, in brown for antigenic site III, in cyan for minor site a, and in green for low pH antigenic site). The location of the 17D2 antigenic site is underlined in pink. Black stars indicate acidic residues in the long helix. Purple stars indicate the other acidic residues or the histidines that play the role of pH-sensitive conformational switches (see text). (TIF) [file ppat.1008383.s003.tif]

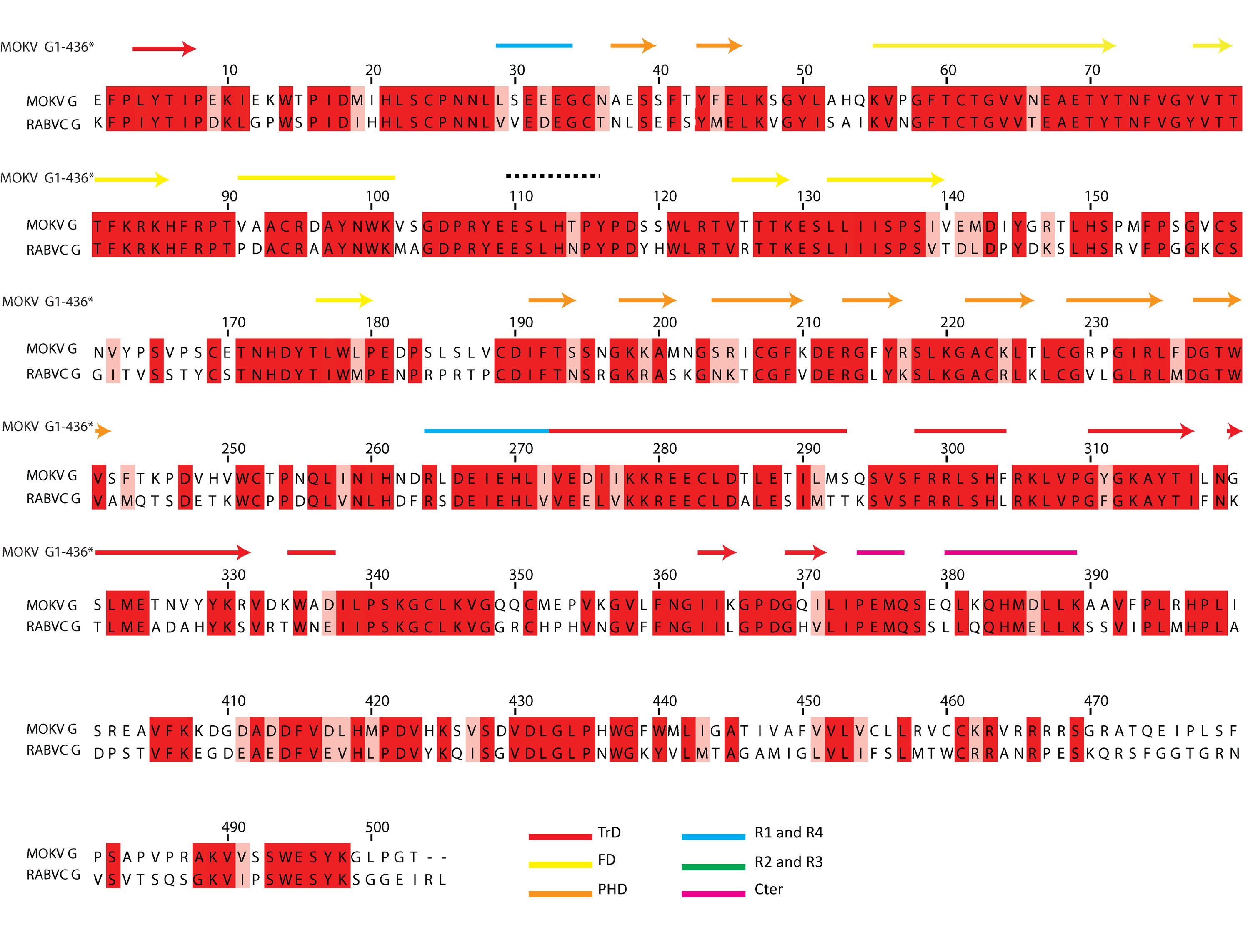

Supplement: S4 Fig — Conserved residues are in red boxes, whereas similar ones are in pink. Colors following Table 2 color code identify domains. Secondary structures of the crystalline MOKV G post-fusion monomer are indicated by arrows for β-sheets and straight lines for helices. (TIF) [file ppat.1008383.s004.tif]

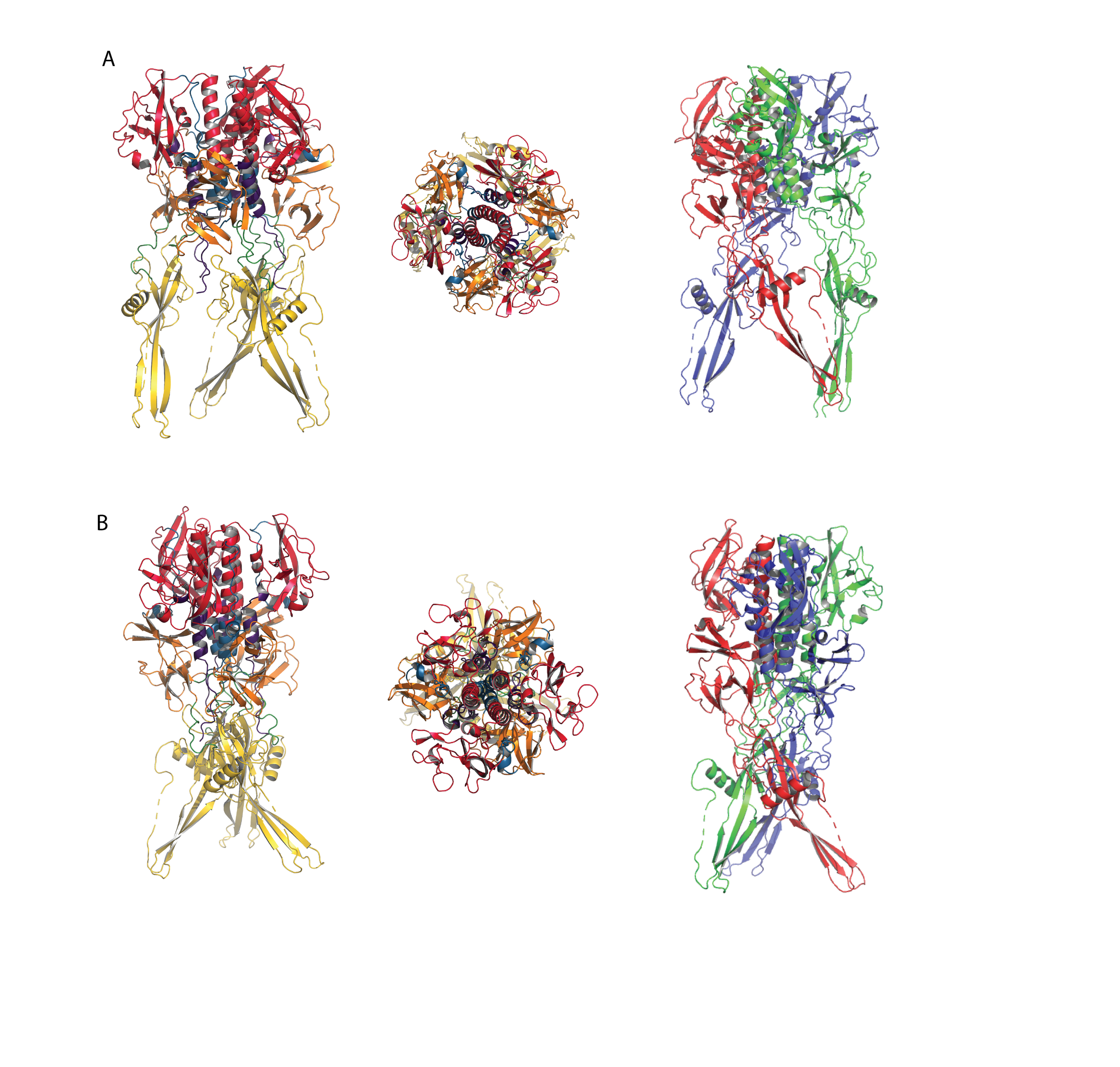

Supplement: S5 Fig — A) MOKV G post-fusion trimer was constructed by modeling the arrangement of the helices at the trimeric interface. For this purpose, the helices were superimposed on a GCNt coiled-coil. In this post-fusion trimer model, there are no steric clashes between subunits. The fusion domains slightly split apart, which may not be the case in the real post-fusion trimer (due to flexibility of R2 and R3 segments or of the fusion domain itself). B) MOKVG post-fusion trimer was constructed by superimposing the Trd domains of MOKV G on the VSV G post-fusion trimer. Steric clashes are visible in top view (showing promiscuity of the three central helices) and in side view (showing important clashes between fusion domains). (TIF) [file ppat.1008383.s005.tif]

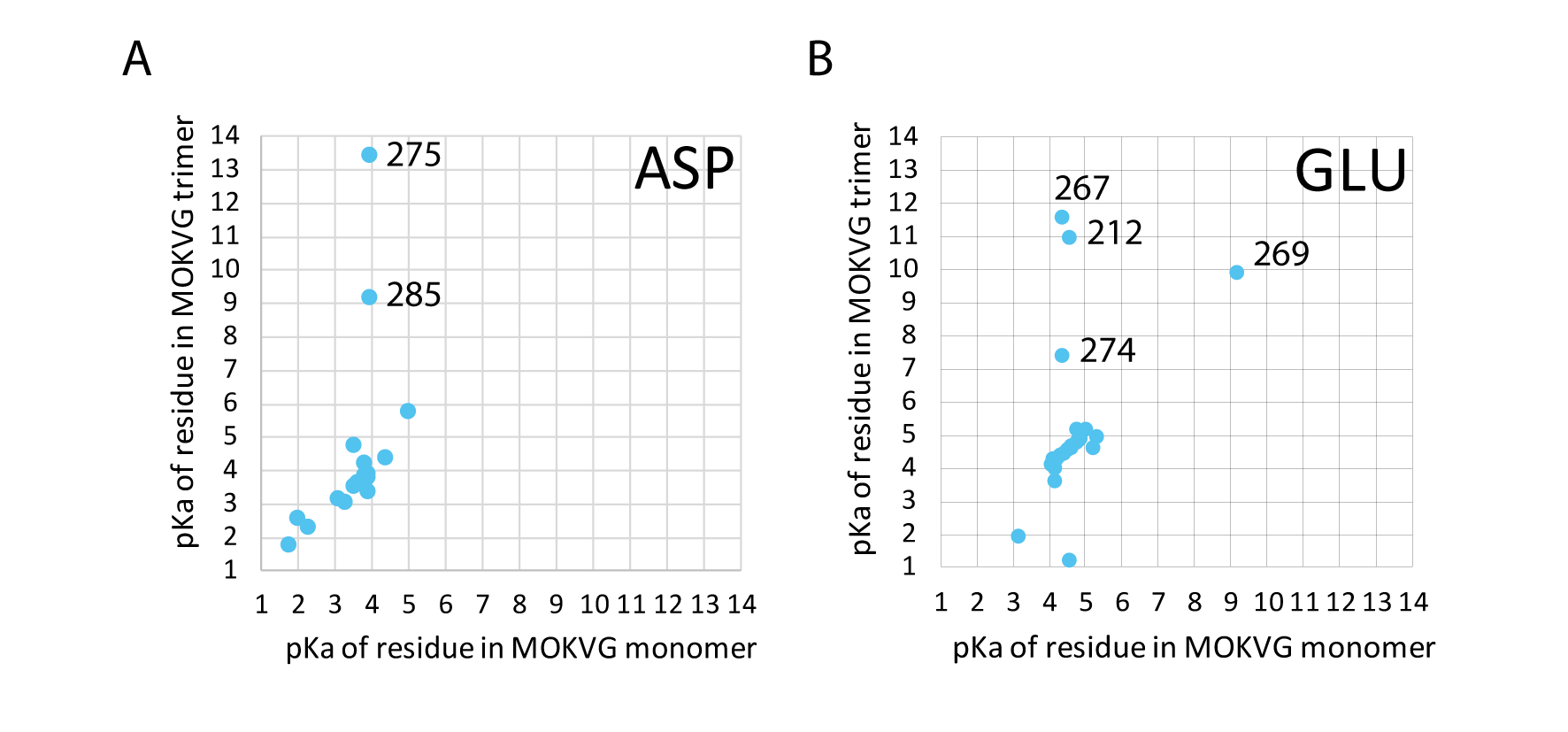

Supplement: S6 Fig — computed pKas of aspartic (A) and glutamic (B) residues in the context of MOKV G monomer plotted against their computed pKa in the modeled post-fusion trimer. Residues exhibiting an important shift (compared to the pKa of Asp and Glu exposed to solvent are numbered). The pKa increase for residue Glu 212 is due to the fact that, in the modeled trimer, it faces Asp 386 of the neighboring protomer. (TIF) [file ppat.1008383.s006.tif]
