# Supplementary material for: Crystal structure of Mokola virus glycoprotein in its post-fusion conformation
Source: PLoS Pathog. 2020 Mar 9;16(3):e1008383. doi: 10.1371/journal.ppat.1008383 (PMC7082061; doi:10.1371/journal.ppat.1008383)
Supplement: Attachment — Submitted filename: DEF Rebuttal.docx [file ppat.1008383.s007.docx]

**Reviewer #1**: Although rabies virus and emerging additional pathogens of the lyssavirus genus, native structural information for the viral glycoprotein is currently available only for members of the related visiculovirus genus (VSV and Chandipura virus) but not for any lyssavirus. This manuscript by Belot and colleagues makes an important first contribution to closing this knowledge gap, reporting the structure of a modified Mokola virus G protein monomer in postfusion conformation. Data are overall of good quality and appropriately presented. A strength of the work is the inclusion of a recombinant VSV-delta G pseudotyped with either wild type or equally fusion loop-modified MOKV G, supporting that the fusion loop modification does not interfere with G trimerization.
This work advances the field, but does not benefit from, or live up to, the repeated overreaching translational claims of “[paving] the way for the development of a pan-lyssavirus vaccine” or use of RABV G “for transneuronal tracing and studies of monosynaptic connections”. These unsubstantiated statements distract from appreciation of the true impact of the work and need to be removed, or appropriately clarified.

**Reviewer #2**: This paper describes the first structure of a lyssavirus glycoprotein, which is important for the development of a pan-lyssavirus vaccine. Overall, the paper reads well and provides a significant contribution to the field.
I invite the authors to address my comments detailed below:

**Reviewer #3**: Crystal structure of Mokola virus glycoprotein in its post-fusion conformation. PPATHOGENS-D-19-02259
This paper by Laura Belot et al presents the first lyssavirus glycoprotein structure in its post-fusion conformation. The work is very well conducted, the experiments nicely designed and undertaken. The I2BC laboratory is and expert team on solving Rhabdoviruses G protein structures and they use their art skills to underpin interesting features about the MOKV G protein structure presented: localization of the antigenic sites, post-fusion trimers, pH sensitive molecular switches.

*We thank the reviewers for their constructive comments. Following are our answers to their comments.*

**Part II – Major Issues**

**Reviewer #1:**

1) 45-46; 61-62; 340: These statements need to be toned down, or substantiated. This structure provides an appreciation of antigenic sites (nicely discussed), but even three-times repetition does not change the fact that “paving the path” is too strong. Antigenic site mapping is based on repositioning of individual MOKV G structural domains on prefusion VSV G, which appears to be a structure guided form of homology modeling (see comment #5), but homology modeling nevertheless. The only actual discussion of the pan-vaccine idea reaches the conclusion that the FD is highly conserved, but makes a poor antibody target (296-299). Although this is certainly correct, this knowledge predates this study and the MOKV G structure adds very little, if anything, to solve the pan-lyssavirus vaccine problem.

*In the revised version, we no longer refer to the panlyssavirus vaccine neither in the abstract nor in the author summary.*

- *In the abstract, the sentence “Globally, this first lyssavirus G structure paves the way for the development of a pan-lyssavirus vaccine” has been replaced by “Finally, the present structure will be a great help to perform rational mutagenesis on lyssavirus glycoproteins”.*
- *In the author summary, we replaced the two last sentences by “This first lyssavirus glycoprotein structure will be useful for teams working on entry and antigenicity of the highly pathogenic viruses of this genus.”*
- *The last paragraph of the discussion has been toned down, we now write: “In conclusion, this work describes the first structure of a lyssavirus glycoprotein, which paves the way for rational mutagenesis. By delimiting structural domains and antigenic sites, it should help to design chimeric lyssavirus glycoproteins, which may be useful for the development of a pan-lyssavirus vaccine.”*

2) 136: Please back up the statement re purity of the protein preparations, i.e. by showing total protein stains after SDS-PAGE.

*A gel showing the purity of MOKV G1-436* and MOKV G1-436WT after affinity chromatography is now shown in Supplementary figure S1.*

3) Can you support your statements in the paragraph starting at line 200 (Fig 5) with 2D class-averaging?

*We tried to perform 2D class averaging using the CryoSPARC software. However, due to the heterogeneity of G staining and the resulting contrast (even at the surface of the same virion), this turned out to be extremely difficult.*

*We have now carefully measured 50 MOKV G spikes having this characteristic shape and found an average length of 11.97 +/- 0.53 nm and a width of 5.3 +/- 0.5 nm, which is typical of VSV G post-fusion trimer length and much wider than MOKV G crystalline monomer (3.5 nm). The text has been modified to account for these observations, which are consistent with MOKV G being in its post-fusion trimeric state (Cf lines 203-208).*

4) 319-320: The GCN4-based postfusion trimer model predicts protonation of at least six acidic residues in the central helix, requiring a pH drop to 5.5-6.0 to be sufficient to result in mass-protonation of aspartate and glutamate side chains at positions 263, 267, 274, 281, 285, and 288. Please provide some detail on the basis and nature of the proposed pKa shift, and add simulations to predict the protonation states of these residues at the appropriate pH points (5.5 and 7.5).

*We used the PROPKA server to compute pKa values of aspartate and glutamate residues in the monomeric crystalline structure and in the modeled post-fusion trimer. This reveals:*

- *a pKa value for E269 (facing D211 in the post-fusion monomer) above 9*
- *a large increase of the pKa values for residues D275, D285, E267 and E274 in the modeled trimer compared to the monomeric form.*

*Those new data are now presented by a plot in Supplementary figure S6 and in the results section (lines 287-294). We have also modified the dedicated paragraph in the discussion (lines 335-340).*

5) 310: There is insufficient data support for this claim. Take multiple measurements, state the number measurements performed, and add class averaging.

*See our answer to comment 3.*

6) The tubular structures shown in figure 4B are not very informative as presented. Can you perform helical reconstructions to determine the regular parameters of these helical arrays?

*We now present cryo-electron microscopy images of those tubular structures. The diffraction pattern reveals that they display helical symmetry (Figure 4C and text lines 192-194).*

**Reviewer #2:**

**Reviewer #3:**

1) The sentence “This work paves the way for the development of a pan-lyssavirus vaccine. Furthermore, as RABV G is used for transneuronal tracing and studies of monosynaptic connections, the knowledge of MOKV G structure might be useful to improve the available tools in this field” is highly speculative at this stage since no experimental data are provided to sustain. Please remove this from the author summary.

*This part has been modified (see answer to major issue 1 raised by reviewer #1).*

2) Identically, the sentence “Globally, this first lyssavirus G structure paves the way for the development of a pan-lyssavirus vaccine” is a hope at this stage.
Both sentences 1) and 2) could be discussed further in the discussion.

*This part has been modified (see answer to major issue 1 raised by reviewer #1).*

3) The authors used two G constructs G(1-436)WT and G(1-436)**. They state that G(1-436)WT was less expressed and its purification was less optimal. Anyhow, they use it for the liposomes experiments. Please provide all the data for the expression and the purification for both constructs.

*A gel showing the purity of MOKV G_1-436_* and MOKV G_1-436_WT after affinity chromatography is now shown in Supplementary figure S1.*

4) Fig 1C is difficult to read. Can the authors increase the quality and/or the magnification?

*We have increased the magnification of the images presented in panel 1C. It is also possible that the figure quality in the originally submitted pdf version was not optimal.*

5) Line 150: “We screened several crystallization conditions….” Please provide more details in the experimental procedures.

*More details have been added in the Material and methods section (lines 451-453).*

6) Paragraph line 180: the authors use G(1-436)WT for the liposomes experiments but they did not try to crystallize it. Could they comment their choice? Having solved the structure of G(1-436)**, don’t they think that some information on G(1-436) could be useful to strengthen their work?

*The fusion loops or lyssaviruses G are much more hydrophobic than those of vesiculoviruses G. We thought that this may limit the solubility of MOKV G ectodomain and replaced the fusion loops by a more hydrophilic motif. Of course, we also tried to express MOKV G ectodomain having the wild type fusion loops. As anticipated, this construct was less soluble than the mutated one. The production yield was also lower. Furthermore, we were unable to get crystals with this construction. However, MOKV G_1-436_WT proved to be useful to study G-liposomes interactions.*

7) To investigate further the biological activity of MOKV G protein, the authors used the VSVΔG-eGFP virus. It is certain interesting to show the pseudotyping feasibility between vesiculovirus and lyssavirus but in this case RABVΔG would have been much more informative. Especially since they observed that the pre-fusion state of G(1-436)** is partially destabilized. I would suggest that experiments with RABVΔG are provided.

*In our team, we do not have the RABVΔG virus. In this work, we have obtained VSVΔG-eGFP pseudotypes that exhibit at their surface WT MOKV glycoproteins having characteristics similar to those previously observed with RABV G at the surface of rabies virions. Furthermore, the work of Piccinotti and colleagues (*J Virol*. 2013.* ***87:****11637-47;* PLoS Pathog. *2016.* ***12:****e1005753) indicated that RABV G is efficiently incorporated in VSV virions and that the resulting particles have properties that are similar to those of RABV ones. Therefore, we do not think that using RABVΔG virus would have been much more informative.*

*The pre-fusion state of G_1-436_* is indeed partially destabilized. We have no explanation for that apart from the fact that the structure of VSV G pre-fusion conformation suggests that the fusion loops might interact with the viral membrane (which is no more possible with the mutated loops).*

8) S2 Fig, even though comparison of all the lyssavirus is interesting, most of the Mab work has been undertaken with RABV. Please provide also a fig with MOKV and RABV only for clarity.

*This alignment is now presented in S4 figure..*

9) Line 300: only P75NTR is discussed. Please add the other receptors in the discussion as far as it is possible.

*We have mentioned the other receptors at the end of the paragraph (lines 320-324). We did not mention them initially because for those receptors, no residue, which would be critical for binding, has been identified in RABV G so far.*

10) Paragraph line 339: According to point 1) authors should discuss further how their structure can help on these matters.

*We have largely suppressed the reference to a pan-lyssavirus vaccine. We conclude now by saying that: “This work describes the first structure of a lyssavirus glycoprotein, which paves the way for rational mutagenesis. By delimiting structural domains and antigenic sites, it should help to design chimeric lyssavirus glycoproteins, which may be useful for the development of a pan-lyssavirus vaccine.”*

**Part III – Minor Issues: Editorial and Data Presentation Modifications**
Please use this section for editorial suggestions as well as relatively minor modifications of existing data that would enhance clarity.

Reviewer #1: 7) 93: “Wavy” does not come across as the best choice of descriptor. I wonder whether you can rephrase the description of the virion surface.

*We have replaced wavy by jagged. “At low pH (below pH 6.4), a jagged layer of elongated spikes (~11.5 nm) in their post-fusion state is observed.”*

8) 222: How was this “repositioning” performed specifically? This strategy appears to be absent from Methods, and without a detailed understanding of the approach this section is not fully convincing.

*The model of MOKV G pre-fusion protomer was obtained by structural superposition of MOKV G FD, TrD and PHD on VSV G pre-fusion protomer (PDB code: 5I2S) using the align command in PyMOL. This is now added at the end of the “Material and methods” section.* *We have also described in this section how we modeled MOKV G post-fusion trimer (lines 480-486).*

9) Please discuss how and why the remarkable diversity of the TrD domain may have evolved. A whole panel (figure 3C) is dedicated to this observation, but discussion of the point remains scarce.

*Figure 3C corresponds to the PHD domain, which indeed is the most divergent domain when MOKV G is compared to vesiculovirus G. The origin of this structural diversity is discussed in the first paragraph of the discussion (lines 301-311). The PHD and the most external part of the TrD which contain antigenic site II and III are not conserved indicating that the humoral response is at the origin of the diversification of rhabdovirus glycoprotein architecture.*

10) 609: Please add a postfusion trimer model based on VSV G and substantiate the claimed “sterical clashes” in a side-by-side comparison in figure S3 to illustrate the point for the reader.

*This is now added in supplementary S6 figure and commented in its legend.*

11) The manuscript needs language editing throughout.

*The manuscript has been corrected by a native English (Scribber company).*

**Reviewer #2:**

Abstract and Results (Line 281): “At high pH, when they are negatively charged, they destabilize the interface, which explains the conformational change reversibility”.
What does mean ‘at high pH’? the pKa of Asp and Glu is ~4.75. These residues will be negatively charged at pH > 4.7. Clarify what ‘high’ pH stands for here.

*See our answer to major comment 4 of reviewer 1.*

Line 109: “Sequence alignments indicate that lyssaviruses glycoproteins adopt the same fold as
other rhabdovirus glycoproteins and therefore belong to the third class (class III) of viral fusion glycoproteins”.
Rephrase. Sequence alignment cannot ‘indicate’ the fold of a protein. Refer instead to sequence similarity and identity.

*The text has been changed.*

Line 134: I find the nomenclature used in this paper (MOKV G1-436**) with two asterisks to be awkward. Perhaps the two asterisks can be replaced with something else.

*The two asterisk were there to indicate that we had modified the two fusion loops. We have now replaced MOKV G1-436** by MOKV G1-436*.*

Line 311: “lyssaviruses and vesiculoviruses, the trimer is unstable”.
Can the authors describe why the trimer is unstable?

*The unstability of rhabdovirus G trimer has been largely described in the literature. However, we have rewritten this passage “This indicates once again that for both lyssaviruses and vesiculoviruses, the trimer is less stable than class I fusion glycoproteins, as largely documented in the literature” (lines 325-328).*

Figure 1C. Right panel. VSV Gth at pH 5.5. Can the authors rule out this protein is not just an aggregate commonly seen by negative stain?

*These are the classical rosettes observed with VSV glycoproteins in their post-fusion conformation when they associate through their fusion loops (See Figure 6 in Albertini et al. PLoS Pathogens 8:e1002556 (2012)).*

Figure 2. It is difficult to decipher what is new (and presented for the first time in this paper) from previously solved structures. Please, dedicate Figure 2 to describe the structure of MOKV G1-436**, then use another figure, or subpanels of Fig 2 to compare MOKV G1-436** to known fusion proteins. Refer to the sequence similarity/identity and RMSD after secondary structure superimposition of MOKV G1-436** with other viral glycoproteins. Also, describe the surface potential, charge, etc. of MOKV G1-436**.

*We have changed Figure 2 according to this remark.*

Figure 7. The paper lacks a 3D-model for the trimeric lyssavirus glycoprotein. Can this be generated using the structural information provided in the work?

*The trimeric post-fusion state was generated and was presented in supplementary figure S4 (now S6). It is too speculative to generate a model of the pre-fusion trimer at this stage.*

Crystallographic table: The I/SI in the outer shell is very low: 0.95, which means the error exceeds the measurement. I find it hard to believe the CC1/2 in the outer shell is 0.798. Also, please indicate the CC1/2 as 0.998 (0.798). Finally, include an Rsym.

*As crystals of MOKV G were anisotropic, data were submitted to the STARANISO server (*[*http://staraniso.globalphasing.org/cgi-bin/staraniso.cgi*](http://staraniso.globalphasing.org/cgi-bin/staraniso.cgi)*) to apply an anisotropy correction of the data. These corrected data were then used for the refinement. This explain why C1/2 is high and the I/SI low in the outer shell.*

*Also As published in Karplus and Diederich (Assessing and maximizing data quality in macromolecular crystallography. Curr Opin Struct Biol. 2015 Oct;34:60-8. doi: 10.1016/j.sbi.2015.07.003), CC1/2 alone assesses the statistical significance of data, and Rsym should play no role in determining the resolution cutoff.*

*The table 1 has been corrected.*
